# Temporal Experience in Addiction and Recovery: A Scoping Review and Conceptual Synthesis of First-Person and Professional Accounts

**DOI:** 10.3390/jcm15218421

**Published:** 2026-10-29

**Authors:** Albert Łukasik, Ryan Kemp, Marcin Moskalewicz

**Affiliations:** 1 Department of Cognitive Science, Nicolaus Copernicus University, 87-100 Toruń, Poland; 2 Central and North West London NHS Foundation Trust, London NW1 3AX, UK; ryan.kemp@nhs.net; 3 Department of Social Sciences, Poznan University of Medical Sciences, 61-701 Poznań, Poland; moskalewicz@gmail.com; 4 Department of Philosophy, Maria Curie-Skłodowska University, 20-031 Lublin, Poland; 5 IDEAS Research Institute, 00-072 Warsaw, Poland

**Keywords:** phenomenology, phenomenological psychopathology, lived time, language, temporality, addiction, substance use, psychotherapy

## Abstract

Phenomenological psychopathology regards disturbed temporal experience as central to addiction, yet most research measures time perception and time perspective with behavioral or psychometric tools, leaving lived experience underexamined. Patients’ descriptions of time have not been systematically compared with those of researchers and clinicians. This scoping review mapped descriptions of temporal experience in addiction and recovery and compared first-person with professional accounts. Following JBI guidance and PRISMA-ScR, we searched PubMed, Scopus, and Google Scholar (November 2023–February 2024; no date limits) for English-language publications (and one Polish-language publication) of any design on temporal experience in substance or behavioral addiction. Of the 623 records, we included 59 publications (1969–2023). We inductively coded temporal terms by perspective (first-person/professional), dimension (past/present/future), and stage (addiction/recovery). No formal quality appraisal was performed. In active addiction, accounts described a narrowed, repetitive present organized around use, a fragmented past, and a foreshortened future. Recovery accounts described renewed access to the past, a more structured present and a wider, more concrete future, with first-person language increasingly resembling professional terms (e.g., “rebuilding”, “continuity”). Evidence came predominantly from substance use disorders. The literature suggests present-focused temporal constriction in addiction and temporal re-integration in recovery. The apparent convergence of patient and professional vocabularies is a hypothesis requiring longitudinal testing before clinical use as a marker of recovery.

## 1. Introduction

### 1.1. Current Research on Time in Addiction

Disturbances in the experience of time are recognized as a feature of many psychopathologies, including addiction [1]. Research on time in addiction has developed along three largely separate lines. Cognitive and neuroscientific studies measure time perception and temporal decision-making, showing, for example, impaired interval timing in stimulant users [2] and steep discounting of delayed rewards in gambling and substance use disorders [3,4,5]. Research on time perspective, most prominently within Zimbardo and Boyd’s framework [6], associates substance use with a dominant present-hedonistic and a weak future orientation [7]. Phenomenological psychopathology, in turn, describes addiction as a disturbance of lived time, that is, of how past, present and future are experienced and structured from within [8,9]. The first two traditions characterize time from the outside, through behavioral tasks and questionnaires; they say little about how people with addiction themselves experience and articulate time, which is the concern of the third tradition [8,10].

Among time perspective frameworks, Zimbardo and Boyd’s theory is the most widely used and provides the temporal structure adopted in this review. Time perspective is defined as the often non-conscious process by which the continuous flow of personal experience is assigned to past, present and future frames that give it coherence and meaning [6]. The Zimbardo Time Perspective Inventory (ZTPI) operationalizes five such orientations: past-negative, past-positive, present-hedonistic, present-fatalistic, and future [6]. Substance use has repeatedly been associated with a high present-hedonistic and a low future orientation [7,11,12], whereas a balanced profile, in which no single frame dominates, has been proposed as adaptive [6]. The theory is relevant to the present study in two ways. First, its division of experience into past, present and future organizes our synthesis, and its findings supply expectations against which first-person accounts can be read: a dominant present in active addiction and a strengthened future in recovery. Second, time perspective is assessed as a dispositional attitude toward time, rated on questionnaire items, rather than as the way time is lived. Whether the pattern captured by ZTPI scores corresponds to how people with addiction describe their own past, present, and future has received little attention, and this is the question a phenomenological approach is suited to address.

### 1.2. Key Concepts and Scope

Several related terms are used in this literature, and we use them as follows. Time perception refers to estimating and discriminating durations, typically measured in timing tasks [2]. Time perspective refers to a dispositional orientation toward the past, present, or future, typically measured with questionnaires such as the ZTPI [6]. Temporal experience, or lived time, refers to time as experienced from a first-person perspective: its felt pace and flow, and how the present connects to what has been and what is to come [8,13]. Temporality denotes the overall structure that integrates past, present and future within experience and underpins a continuous sense of self [9]. We use temporal dysregulation as an umbrella term for any departure from this integrated structure described in the sources, such as a narrowed or “frozen” present, an inaccessible past or a foreshortened future, and temporal integration for the re-establishment of connections between the three dimensions. Temporal disintegration, a term used in some phenomenological sources, is treated here as the specific loss of continuity between dimensions and thus as one form of dysregulation.

The review compares two perspectives on temporal experience. First-person accounts are descriptions given by people with addiction or in recovery in their own words, usually reported as verbatim quotations in interviews and case studies. Professional accounts are the terms and concepts researchers and clinicians use to describe or interpret these experiences, whether derived from interviews, therapeutic work, psychometric data, or phenomenological analysis [10]. Researchers who work directly with first-person material occupy an intermediate, second-person position; Section 2.6 describes how we handled this analytically.

Addiction is understood here in line with current diagnostic classifications, which group substance use disorders with gambling disorder based on shared features such as impaired control, craving, and continued engagement despite harm [14,15]; ICD-11 additionally recognizes gaming disorder [15]. We include both substance and behavioral addictions because the temporal features of interest, most notably a preference for immediate over delayed rewards, have been reported in both [3,4,5]. Because the included sources used different criteria (formal diagnosis, treatment status, or self-identification), we adopted each source’s definition; wherever a finding concerns a particular substance or behavior, we indicate this in Section 3. Recovery is understood as a voluntarily maintained lifestyle characterized by sobriety, personal health and citizenship [16], and thus as a process rather than a single endpoint, one that may include relapse. Operationally, accounts were assigned to the recovery stage when they described people in treatment, either abstinent or self-identifying as in recovery, and to the addiction stage when they described active use or the period preceding treatment.

### 1.3. Research Gap, Aim and Questions

Although these three traditions have each advanced the understanding of time in addiction, they have rarely been brought together. Studies of time perception and time perspective show that temporal orientation differs in addiction, but not in how time is actually experienced [2,3,4,5,6,7,11,12]. Phenomenological and qualitative studies offer rich first-person accounts. Still, they are dispersed across disciplines, use heterogeneous and often idiosyncratic vocabularies, and have largely been read one study at a time [8,10]. To our knowledge, no review has compared the language in which people with addiction describe their temporal experience with the language researchers and clinicians use to interpret it, or examined how either differs between active addiction and recovery. This gap has clinical relevance: when patients and professionals describe the same experience in different terms, shared understanding in treatment may be impeded, whereas points of convergence may indicate where clinical concepts capture what patients live through.

This scoping review therefore aimed to map how the literature describes temporal experience in addiction and recovery and to compare first-person with professional descriptions. Using the division of experience into past, present and future as an organizing structure (Section 1.1), we addressed two questions: (1) How do people with addiction describe their past, present and future, and how do these descriptions compare with the terms used by researchers and clinicians? (2) How do these descriptions differ between active addiction and recovery? The review contributes a structured map of temporal descriptions across first-person and professional sources; a shared vocabulary linking phenomenological, psychological and clinical terms; and hypotheses about temporal change in recovery that can be tested in longitudinal research.

## 2. Materials and Methods

### 2.1. Study Design

This study is a scoping review followed by a conceptual synthesis. A scoping design was chosen because the aim was not to estimate an effect or to aggregate the findings of methodologically homogeneous studies, but to map how temporal experience in addiction and recovery has been described across the heterogeneous literature (qualitative interview studies, phenomenological and theoretical analyses, psychological, including psychometric, studies and intervention reports) and to clarify the concepts used to describe it. Clarifying key concepts and identifying knowledge gaps are recognized indications for a scoping review [17]. The review followed the Arksey and O’Malley framework [18], as refined by Levac et al. [19] and the JBI methodology for scoping reviews [20], and is reported in accordance with the PRISMA Extension for Scoping Reviews (PRISMA-ScR) [21]. The completed PRISMA-ScR checklist and the PRISMA 2020 checklist [22] are provided in Supplementary Tables S1 and S2. No review protocol was prepared or registered. After the mapping stage, we synthesized the charted descriptions conceptually (Section 2.6). Because the included sources differ in design, we treat them as sources of descriptions and concepts of temporal experience rather than as evidence of equal strength, and we present the resulting framework as hypothesis-generating rather than as a validated model.

### 2.2. Eligibility Criteria

Eligibility was defined using the Population–Concept–Context framework recommended for scoping reviews [20]:

- Population: This refers to people with a substance use disorder or a behavioral addiction (e.g., gambling), of any age, and at any stage from active addiction to recovery. Because the literature used different criteria, we accepted addiction and recovery as defined in each source (formal diagnosis, treatment status, or self-identification; see Section 1.2); a formal diagnosis was not required.
- Concept: This includes descriptions of temporal experience relating to the past, present, or future, either in the words of people with addiction (first-person accounts) or in the terms used by researchers and clinicians (professional accounts). Studies of time perception and time perspective were eligible when they characterized how time was experienced or oriented, not only when they reported a performance score.
- Context: This includes any setting, including treatment services, the community, and prisons.
- Types of sources: This includes empirical studies of any design (qualitative, quantitative, and mixed methods), reviews, and theoretical or phenomenological papers, including scholarly book chapters, with the full text available in English and no restriction on publication date; one Polish-language source, read by the first author (a native speaker), was also included.

We excluded sources that did not concern addiction, did not describe temporal experience, or were not available in English or Polish. We grouped included sources for synthesis by perspective, temporal dimension, and stage, as described in Section 2.6.

### 2.3. Information Sources and Search Strategy

We searched three sources: PubMed, Scopus, and Google Scholar. We searched from November 2023 to February 2024, with no publication date restriction. Rather than a single Boolean string, the search used a series of free-text keyword combinations in which a population term (addiction, addicted, or substance abuse) was combined, using the operator AND, with one or more terms for time and temporal experience (time, time perception, time flow, lived time, synchrony, temporal*, temporal experience) and, in the more specific combinations, with a term denoting a phenomenological or first-person perspective (phenomenology, phenomenolog*, first-person). We enclosed multi-word terms in quotation marks and used an asterisk for truncation. In PubMed and Scopus, we restricted searches to titles, abstracts, and keywords; in Google Scholar, which does not allow this restriction, we searched with its default settings. We used only free-text terms, without controlled vocabulary (e.g., MeSH), and applied no search filters. Twenty-eight keyword strings were run in each source, giving 84 query–source combinations, of which 81 returned at least one record. Broad combinations (e.g., addiction AND time, which returned more than 20,000 records in PubMed) were used to calibrate the search and were not screened record by record. We screened records retrieved by the 22 combinations marked in Supplementary Table S3: 10 combinations in PubMed (224 records) and 11 in Scopus (299 records). Because Google Scholar ranks results by relevance and does not allow complete export, we screened the first 10 pages (100 records) of the combination addiction AND time* AND phenomenolog*; later pages contained predominantly non-English, duplicate, or irrelevant records. In total, we identified 623 records. An example search string (PubMed) is as follows: addiction AND time* AND phenomenolog*. Supplementary Table S3 provides the complete list of keyword combinations and the number of records retrieved from each source.

### 2.4. Selection of Sources

**Figure 1:**
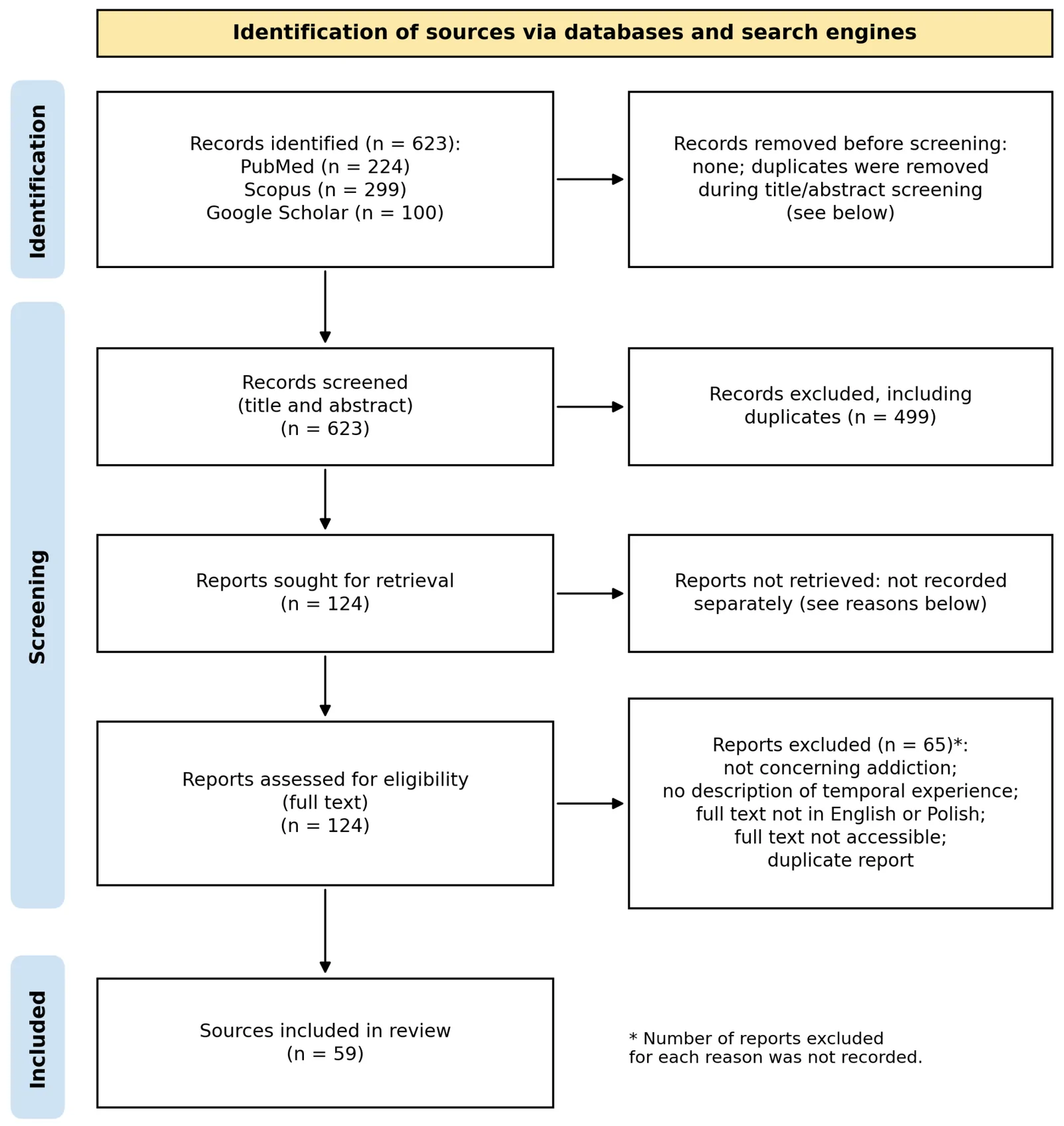
PRISMA flow diagram of source selection. Duplicate records were removed during title and abstract screening and are included in the number of records excluded at that stage.

A single reviewer (A.Ł.) selected sources in two stages and discussed uncertain cases with a second author (M.M.) until agreement was reached. In the first stage, we screened the titles and abstracts of 623 records against the eligibility criteria (Section 2.2). We removed duplicate records, including identical result sets that PubMed returns for “addiction” and “addicted”; because they were not counted separately, we included them in the 499 records excluded at this stage. We sought the remaining 124 reports for full-text assessment. Of these, we excluded 65 because they did not concern addiction, did not describe temporal experience, were not available in English or Polish, could not be accessed in full text, or duplicated an included report; we did not record the number excluded for each reason. In total, the review included 59 sources. No automation tools were used. The PRISMA flow diagram (Figure 1) summarizes the selection process.

### 2.5. Data Charting

One author (A.Ł.) charted the data in Mendeley Reference Manager (version 2; Elsevier, London, UK), recording terms describing temporal experience as tags for each source. For each included source, we recorded the author(s) and year of publication, the type of source, the study design, the type of addiction (substance or behavior), the population and setting, and whether the source contributed first-person accounts, professional concepts, or both, relating to the addiction stage, the recovery stage, or both. Supplementary Table S4 summarizes these characteristics.

To describe the heterogeneity of the literature, each source was also classified according to its main approach: (1) intervention studies, focused on changes in temporality brought about by treatment and described in terms such as “new projection of self” [10], “augmented future” [23] or “re-evaluated past” [24]; (2) psychological studies, in which temporality was examined mainly with tests and questionnaires, such as the ZTPI [11]; (3) interview studies, which collected personal accounts of temporal experience from people with addiction, such as having “no sense of time at all” [25]; and (4) phenomenological studies, which offered theoretical accounts of the temporality of addiction. Table S4 reports this classification for each source, but we did not use it as a unit of analysis. Because all four groups described the same temporal phenomena, we organized the synthesis by perspective, temporal dimension, and stage (Section 2.6).

Consistent with JBI guidance for scoping reviews, we did not formally appraise individual sources [20]. The aim was to map how temporal experience is described, not to estimate the strength of evidence for an effect; moreover, a substantial proportion of the included sources are theoretical or phenomenological analyses to which no appraisal tool applies. To avoid treating heterogeneous sources as equivalent evidence, the synthesis distinguishes first-person accounts from professional interpretations throughout.

### 2.6. Synthesis

The synthesis combined inductive and deductive steps, in line with recommendations for basic qualitative content analysis in scoping reviews [26].

First, each included source was read in full, and every term or phrase describing temporal experience was recorded as a tag in Mendeley, retaining the wording of the source (e.g., “atemporalization”, “fracturing of time”, “blackout”, “out of sync”, “the now”, “desynchronization”, “lost time”, “spillage of time”). This yielded more than 100 tags. The vocabulary differed by type of source: interview participants described being “trapped” in the present; intervention studies referred mainly to changes brought about by therapy, such as “memory reconsolidation”; psychological studies used established constructs and questionnaire categories, most often from the ZTPI, such as “present-hedonistic time perspective”; and phenomenological papers used metaphorical terms such as “fake future”, “escape from time”, “floating world” or “anti-time”.

**Figure 2:**
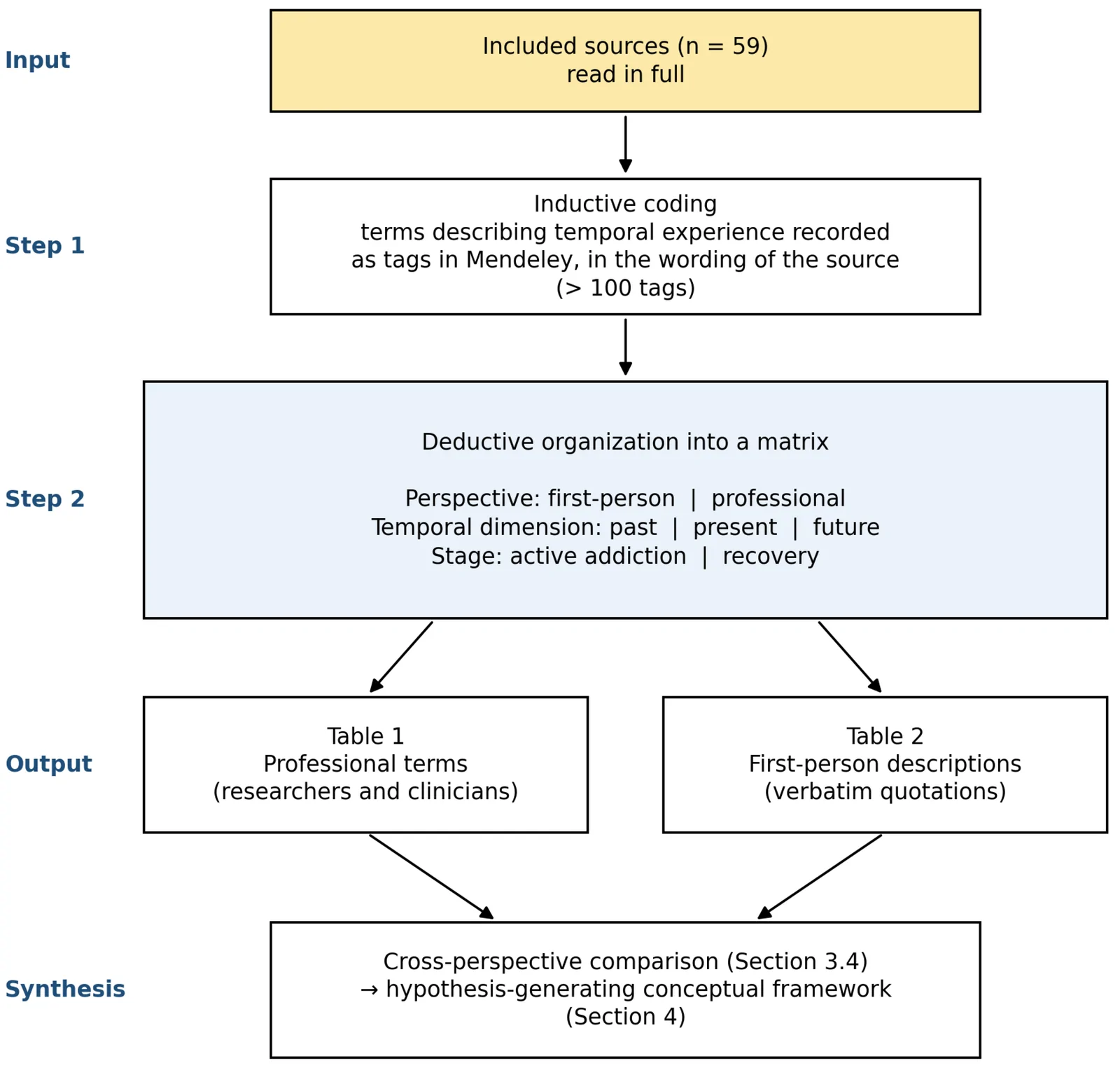
Synthesis process: inductive coding of temporal terms, their organization by perspective, temporal dimension and stage, and the resulting tables and cross-perspective comparison.

Second, to make these heterogeneous vocabularies comparable, the tags were organized in a matrix defined by three criteria derived from the research questions and from time perspective theory (Section 1.1): perspective (first-person or professional), temporal dimension (past, present or future) and stage (active addiction or recovery). We assigned tags to the temporal dimension whenever the source made this possible; the same term could therefore appear under more than one dimension when different sources used it for different dimensions (e.g., “increased emphasis” for both the past and the future). Professional terms and first-person descriptions are summarized in two tables presented in Section 3.1, and Section 3 juxtaposes the two perspectives for each dimension and stage. Figure 2 summarizes the process.

A description was classified as first-person when the source presented it as the participant’s own words, typically as a verbatim quotation (in two cases, a published autobiographical account quoted by the source; marked in Table S5), and as professional when the wording was that of the researchers or clinicians, including their summaries and interpretations of interview material. Researchers who work directly with first-person material occupy an intermediate, second-person position, and their interpretive terms are not equivalent to third-person measures such as questionnaire scores. The professional category therefore spans both, and where the difference matters for interpretation, Section 3 indicates it. Researchers and clinicians were combined in one category because both translate patients’ experience into a conceptual vocabulary, and the synthesis aimed to contrast this vocabulary with patients’ own words.

A single author (A.Ł.) carried out all coding and categorization; no second coder was involved, so inter-rater reliability was not assessed. Because the aim was to map the range of descriptions rather than to develop themes to saturation, saturation was not assessed. The authors approached the material from different backgrounds (cognitive science, phenomenological psychopathology and clinical practice in addiction services). Two authors (R.K. and M.M.) published work on temporality in addiction that is among the included sources; these sources were coded in the same way as all others, and we acknowledge this as a potential source of bias (Section 4.4).

## 3. Results

### 3.1. Included Sources and Structure of the Results

The 59 included sources were published between 1969 and 2023 and comprise interview studies, phenomenological and theoretical analyses, psychological (including psychometric) studies and intervention reports (Supplementary Table S4). Most concern substance use disorders: of the 59 sources, 12 concerned alcohol, six a single other substance (opioids, *n* = 3; stimulants, *n* = 2; cannabis, *n* = 1), 27 drug use involving several or unspecified substances, and 12 addiction in general; behavioral addictions were the specific focus of only two sources (gambling, *n* = 1; internet use, *n* = 1), and one review also covered gambling.

The synthesis is presented first for the addiction stage and then for the recovery stage. For each stage, the past, present, and future are described in turn, and for each dimension first-person descriptions are presented before professional terms. Table 1 lists the professional terms used by researchers and clinicians, and Table 2 lists first-person descriptions, for each dimension and stage. Where a finding comes from a particular substance, behavior, or setting, this is stated. Section 3.2 and Section 3.3 summarize what the included sources report; we reserve our interpretation of these findings for Section 3.4 and Section 4.

### 3.2. Addiction Phase

#### 3.2.1. First-Person Accounts of the Past in Active Addiction

In first-person accounts, the past of active addiction is often described as unavailable because of memory gaps associated with intensive use. Participants report difficulty remembering both the period before addiction and the addiction itself, with recollection reduced to the last occasion on which craving was relieved (alcohol [60,69]). Not all accounts share this pattern: in some, stressful or traumatic past events are recalled as having led to addiction [24,64], and in others the past is recalled as a series of bad decisions (adolescent cannabis users [61]). Some participants remember their pre-addiction self more positively than their present one, although these memories remain vague [73]. Most sources, however, report that participants could not recognize their pre-addiction selves or remembered little of their past, particularly in the context of alcohol-related blackouts [27,32].

#### 3.2.2. Professional Accounts of the Past in Active Addiction

Professional sources describe the relation to the past in terms of “cutting ties” [66], that is, using drugs deliberately to forget traumatic events. This is described as leading either to complete forgetting of the past [27] or to fragmented memory, which may also result from frequent alcohol-induced blackouts [28] and has been reported in gambling disorder [57]. Several authors argue that losing access to the past impairs the narrative self, so that the person cannot locate themselves in time or within their social and personal context [37,41].

#### 3.2.3. First-Person Accounts of the Present in Active Addiction

First-person descriptions of the present are dominated by the need to “fast-forward” time to the next dose [38,63], without regard to past or future actions. Participants report not noticing time passing from day to day, or even from year to year (young drug users [59]). The present is described as cyclic and organized around a simple routine of obtaining the substance, sobering up and obtaining it again [10,75], and as endless and static, stripped of any flow (heroin and cannabis users [47]). Participants describe themselves as suspended in time, with every day consisting of the same actions in a never-ending cycle [59], as in accounts of crack cocaine use [48]. Phenomenological authors summarize this as a present that extends only over the next few hours, rarely over days, weeks or longer [29,42].

#### 3.2.4. Professional Accounts of the Present in Active Addiction

Professional descriptions were conceptually richest for the present. A frequent term was “the now”, denoting being stuck in and focused solely on the present, without extending lived time to the past or future [76]. The term “twilight state” was used to describe drug use as a way of fast-forwarding through conventional time, disrupting the continuity between temporal dimensions (heroin [36]). Philosophical terms such as “anthropological hyperpresentification” [23] and “atemporalization” [46] describe essentially the same phenomenon: an exclusive focus on the present.

#### 3.2.5. First-Person Accounts of the Future in Active Addiction

First-person accounts describe shortened temporal horizons, within which envisioning oneself and planning are markedly impaired. Most interview material shows little consideration of the consequences of continued use; in some accounts, however, the turning point (the decision to stop using or to enter treatment) came from thinking about possible death or problems in the near future [65]. When asked to describe their future in more detail, participants gave descriptions that lacked detail and were dissociated from the present and the past [43]; others could not describe it at all, finding it hard to imagine or to separate from the present state [29]. Participants found it difficult to picture a self without addiction beyond the immediate future [29,43], a pattern that professional sources describe as a “closed” future (Section 3.2.6).

#### 3.2.6. Professional Accounts of the Future in Active Addiction

Professional sources describe fixation on the present as preventing people with addiction from planning beyond the moment or adopting a future perspective [39,49], and as leading to neglect of the future consequences of their actions [30]. This focus may not be entirely intentional: one author describes the present as a “black hole” that pulls people away from past memories and future plans [56]. When future plans are formed, they are reported to be vague and often unfulfilled [5,31]; in heroin users, this has been linked to shortened time horizons and insensitivity to future consequences [5].

### 3.3. Recovery Phase

#### 3.3.1. First-Person Accounts of the Past in Recovery

In recovery, the previously unavailable past becomes partly accessible again (“And I’m remembering things” [63]). Participants report being able to reflect on their past selves and to recognize how earlier decisions led to their current situation [66]. Accounts differ in how far back this reaches: some participants recall the time before addiction, whereas others recall mainly the repetitive cycle of addiction itself [63,66].

#### 3.3.2. Professional Accounts of the Past in Recovery

Whereas professional sources describe the addiction-stage past as fragmented, lost and inaccessible (Section 3.2.2), their recommendations for recovery focus on re-establishing access to it: encouraging patients to “look backward” [25], to reflect on the self before addiction took over, or to construct a “new projection of self” in the past that allows a linear, chronological narrative to extend into the present and future [10].

#### 3.3.3. First-Person Accounts of the Present in Recovery

During treatment, some participants reframed their first drink as a misguided decision that later developed into addiction [77]. Incarcerated participants in recovery described their past self with addiction negatively and their present situation, even in prison, as considerably better; sobriety gave them time to reflect on their actions, because the present was no longer “fast-forwarded” by drug use and craving was no longer their only motive for acting. Some of them also began to see a future on the horizon, which allowed more detailed plans for life after release (Singapore [74]).

#### 3.3.4. Professional Accounts of the Present in Recovery

Professional sources contrast recovery with the addiction-stage present described as “frozen” or “immediate” (Section 3.2.4), but their therapeutic recommendations differ. Some report or recommend a reduced orientation towards the present in favor of the future (treatment samples [51]; alcohol dependence [52]); others propose cutting the present into smaller, achievable portions that gradually extend it (young adults in early recovery [53]); and phenomenological clinicians propose reducing the intensity of present experience while increasing the emphasis on the past and future [33].

#### 3.3.5. First-Person Accounts of the Future in Recovery

In first-person accounts of recovery, the future, previously distant and indefinite, moves closer to the present and becomes easier to envision [54]. As treatment progresses, plans for the future become more detailed and concrete, as in adolescent drug users who described specific educational and career goals [67].

#### 3.3.6. Professional Accounts of the Future in Recovery

In professional sources, the addiction-stage future is characterized as being pulled into the present [51,54], closed to the imagination [24,29,30] or too distant to envision [38]. Therapeutic efforts to change the future perspective are described as “moving forward” [25] and as strengthening orientation towards the future self [38,51]. Accordingly, the future in recovery is described as “augmented” or extended [23,58] and as receiving increased emphasis [24,31,33,49].

### 3.4. Comparison of First-Person and Professional Accounts

Across both perspectives, the included sources converge on a common pattern. In active addiction, the present narrows and repeats, the past fragments or becomes inaccessible, and the future foreshortens or closes; in recovery, the past becomes accessible again, the present becomes more structured, and the future widens and becomes more concrete (Section 3.2 and Section 3.3; Table 1 and Table 2). The two perspectives differ mainly in vocabulary. First-person accounts describe temporal experience through concrete, everyday images and metaphors, such as a “big hole” in memory [59], a “never-ending dream” [48], or “a little time-out” [69]. In contrast, professional accounts use technical and philosophical terms such as “time perspective”, “anthropological hyperpresentification” or “atemporalization” (Table 1).

First-person language also differs between stages. Addiction-stage descriptions are largely confined to the immediate present, whereas recovery-stage descriptions more often explicitly compare the past, present, and future. Some recovery-stage descriptions use expressions that also occur in treatment and recovery-community discourse, for example “just for today” [53,64], “the power of now” and having “learned, via treatment, to live in the present” [68], or describing change as “a process” [34]. We call this overlap an apparent convergence between first-person and professional vocabularies. It is not universal: one participant explicitly distanced himself from the word “recovery” [64].

The present review cannot establish what this convergence reflects. It may indicate a genuine change in temporal experience. Still, it is equally compatible with improved cognitive functioning during abstinence, with exposure to treatment and the adoption of therapists’ vocabulary (as the examples above suggest), with the discourse of recovery communities such as twelve-step groups, or with social desirability in interview settings. Moreover, addiction-stage and recovery-stage accounts came largely from different participants and studies, so the comparison is cross-sectional and does not show change within the same individuals. The convergence should therefore be regarded as a hypothesis to be tested in longitudinal research, not as an established finding.

In our interpretation, the patterns summarized above are consistent with the phenomenological view of addiction as a disturbance of temporal integration. If the past is inaccessible, past consequences such as guilt, shame, or harm may lose their capacity to shape present choices, and the narrative continuity of the self may be weakened; in recovery, reframing earlier decisions (e.g., the first drink as a “misguided decision” [77]) may allow people to see themselves as someone they once were but no longer are, and to build a narrative that leads from past addiction to the present. These links are inferences from the synthesized material rather than findings reported by the included studies.

Change between the two stages is rarely linear. Recovery may involve several attempts interrupted by relapse, and relapse accounts show how vividly the earlier period of use can return (“Time does not forget… I was drunk for 3 months after that” [69]). In such accounts, the renewed ability to envision the future appears to be lost again, together with the return of a present-centered pattern [69].

## 4. Discussion

### 4.1. Principal Findings and Theoretical Implications

This scoping review mapped how temporal experience in addiction and recovery is described across 59 sources. Three findings stand out. First, first-person and professional accounts describe the same overall structure: a narrowed, repetitive present, an inaccessible or fragmented past, a foreshortened future in active addiction, and a gradual reopening of past and future in recovery. Second, the two perspectives differ mainly in vocabulary, with concrete images and metaphors in first-person accounts and technical constructs in professional ones. Third, some recovery-stage first-person descriptions use expressions also found in treatment and recovery-community discourse; whether this reflects a change in experience or the adoption of a vocabulary cannot be determined from the present data (Section 3.4).

These findings are broadly consistent with time perspective theory. The dominance of the present, the negative or fragmented past and the weak future orientation described in active addiction correspond to the present-oriented and low future-oriented profiles repeatedly reported in substance use [6,7,11,12], and the strengthening of future orientation in recovery corresponds to the increase in future time perspective observed during and after treatment [51,52,58]. In theory, recovery can thus be read as movement toward a more balanced time perspective [6].

At the same time, the first-person material points to aspects of temporal experience that a dispositional measure does not capture. First, time perspective scales assess attitudes towards the past, but many accounts describe a past that is not so much negatively valued as unavailable, lost in memory gaps and blackouts [27,32,54,59]; the problem here is access to the past rather than its valence. Second, the present of active addiction is often described not as hedonistic but as compulsive and repetitive (“Sick every day” [63]; getting stoned “so I could go to bed again” [59]), which fits phenomenological descriptions of a “frozen” or “atemporal” present [10,36,46] better than the notion of a present-hedonistic orientation. Third, what changes in recovery is not only the strength of the orientation towards each dimension but the connection between them, and the ability to relate past decisions, present actions and future possibilities [34,66,74]. These observations suggest that time perspective theory and phenomenological psychopathology are complementary: the former describes the direction of temporal orientation and the latter describes its structure and lived quality [8,9].

More generally, the review shows that phenomenological descriptions and empirical findings on addiction can be related within one framework, in line with approaches that combine phenomenological inquiry with clinical and empirical research [10]. The shared vocabulary that emerges from comparing the two perspectives may facilitate communication between patients, clinicians, and researchers. Still, this possibility was not tested in the present review and remains a hypothesis for future work.

### 4.2. Research Gaps

The review identified several gaps in the literature. The most important concern is study design. Almost all included sources are cross-sectional: descriptions of active addiction and of recovery come largely from different participants, so no source traces how the temporal experience of the same individuals changes from active addiction through treatment to recovery (Section 3.4). Longitudinal qualitative studies following the same people before, during, and after treatment are needed to test whether the temporal changes and the apparent convergence of vocabularies described here reflect changes in experience within individuals.

Second, little attention has been paid to how disturbed temporality develops. Most studies aimed to demonstrate temporal dysregulation in established addiction rather than to trace its emergence, from first engagement with a substance or behavior to the most severe stages preceding treatment. Since dose and frequency of use usually increase gradually, temporal changes may also be incremental rather than abrupt, which is a possibility that remains unexamined.

Third, comparisons between age groups are lacking. The age at which addiction develops may be related to cognitive capacities, experience with substances, and social situation, all of which may shape how temporal experience is described. Rodriguez and Smith [53], who studied young men in early recovery, suggest that younger people’s experience of recovery may differ from that of adults, with implications for treatment; adolescents’ accounts were available in only a few sources [61,67].

Fourth, qualitative comparisons between substances are missing. Quantitative studies suggest that temporal alterations differ in part by substance: a review by Paasche et al. [44] reports that alcohol use is associated with a faster retrospective passage of time, whereas steeper discounting of delayed rewards has been found across tobacco, cannabis and cocaine use and in pathological gambling, the latter of which is also associated with shortened time horizons. Qualitative research has not examined whether such differences are reflected in lived experience.

Finally, behavioral addictions are markedly under-represented: only a few included sources concerned gambling [57] or internet use [71] compared with the large number of studies on substance use. Studies comparing substance and behavioral addictions, and studies examining cultural contexts, including differences between countries in the availability of particular substances and activities, would show whether the temporal patterns described here generalize across types of addiction.

### 4.3. Clinical Implications

The clinical implications below are derived from the synthesis and from the clinical experience of one of the authors (R.K.); they have not been tested in the present review and should only be read as suggestions for practice and research.

An important starting point is stigma. Even within treatment services, people with addiction are often described as impulsive, unreliable, or as having a “chaotic lifestyle”, and such behavior is frequently understood in moral terms rather than as an intrinsic component of the condition. Understanding these behaviors as expressions of a disturbed temporal experience may help to counter such moral readings and support a more balanced relationship between clinicians and people seeking help.

To make these implications more concrete, Table 3 relates the temporal patterns identified in this review to four phases of a typical treatment process: assessment and engagement, early treatment and stabilization, psychotherapy, and relapse prevention and aftercare. For each phase, it suggests a clinical focus and examples of practices, drawn from the included sources, through which this focus could be addressed. The suggestions are not a validated protocol; they indicate where attention to temporal experience could be built into existing treatment rather than requiring a separate intervention.

At assessment, clinicians could ask explicitly about each temporal dimension: what the patient remembers of the period before and during use, what an ordinary day currently consists of, and what they expect in a month or a year. The metaphors through which patients describe their relation to time, such as being “trapped”, “stuck” or “lost”, can give clinicians access to the lived reality of addiction. Instruments such as the Transdiagnostic Assessment of Temporal Experience (TATE) [1] or the ZTPI [6] can complement this conversation, and repeating them at discharge would allow services to document change. In early treatment and stabilization, the daily structure typical of residential programs [68] can be presented to patients as a way of rebuilding a present that is no longer organized around obtaining and using the substance, and the present can be divided into short, achievable units, as described by young men in early recovery [53]. Making the temporal rationale of this structure explicit may also reduce the mismatch between the time perspectives of patients and staff reported in treatment settings [49].

In psychotherapy, patients often experience confusion about their sense of self [30], and work on developmental history can be framed as reconnecting the past with the present. This can include re-evaluating past decisions, such as when participants came to see their first drink as a misguided decision [77], and approaches that target the neglect of the past and the dominance of the present [24]. To widen the future, patients can be asked to imagine specific near-future events, including episodic future thinking combined with cue extinction and memory reconsolidation [39], and to set concrete short-term goals, such as the educational and career plans described by adolescents in treatment [67]; mindfulness-based interventions may help patients attend to the present without the compulsive narrowing described in active addiction [78]. In relapse prevention and aftercare, a renewed narrowing of the present and the loss of future plans, as described in relapse accounts [69], can be discussed with patients as early warning signs, and maintaining concrete plans may support continued recovery; temporal orientation has been associated with the course and outcome of treatment [51,52]. Whether changes in how patients describe time could help clinicians recognize progress in recovery remains an open question; given the alternative explanations discussed in Section 3.4, such changes should not be used as a marker of recovery until they have been validated empirically.

By way of illustration, we offer a brief anonymized vignette from the clinical practice of one of the authors (R.K.). It is not research data and was not part of the synthesis: it was not collected for this study; the patient is referred to by a pseudonym (“Joan”); and details that could identify her, such as age, location, and dates of treatment, are omitted. Joan was repeatedly admitted to hospital with infections at injection sites. She said she could not wait for her wounds to heal properly and would remove the dressings and resume injecting: “I do no waiting for weeks and weeks, I have to live today, otherwise I go cold turkies. Don’t lecture me about medical advice. I got street advice”. It took months of work to start Joan on methadone, and longer to stabilize her so that she stopped injecting. Later she said: “In them days I just did what I wanted, when I wanted. I need to score, so I did. Now I have learned about getting structure in my life, about how you get rewards in your life, but not like every day. You cannot be so impulsive all the time, it’s not growed up”. In her account, a few ideas from therapeutic work (structure, delayed reward and impulsiveness) appear to have changed how she related to the present and the future. A single anecdotal case cannot show that such a change occurs in general or what caused it. It is offered only to illustrate how the temporal dimensions described in Section 3 may surface in clinical conversation.

A further, hypothetical illustration concerns a person who comes to accept that there are no immediate solutions to their problems and decides, for example, to look for a volunteering position that may later lead to paid work. The move from “I must find work this week” to “I can take baby steps towards getting back to work, and that is OK” involves a subtle temporal shift from an immediate to an extended horizon.

Finally, although the first-person and professional vocabularies overlap in part, the metaphors used in active addiction convey aspects of temporal experience that technical terms do not. In contrast, therapeutic approaches offer ways of addressing these disturbances. Both perspectives therefore converge on temporality as a possible focal point for intervention and further research.

### 4.4. Limitations

Several limitations concern source search and selection. The search used keyword combinations rather than a single Boolean strategy and did not use controlled vocabulary; relevant studies indexed under other terms may therefore have been missed. Google Scholar results depend on a relevance-ranking algorithm and cannot be fully reproduced, and we screened only the first 100 records. We included only English-language sources (and one Polish source), and we did not systematically search the grey literature, so we cannot rule out publication and language bias. A single reviewer carried out screening, with uncertain cases discussed with a second author, which increases the risk of selection errors. In addition, we did not record the number of full-text reports excluded for each reason during screening, and we could not reconstruct it (Figure 1).

Other limitations concern the evidence base. The included sources are heterogeneous in design, ranging from interview studies to psychometric studies and theoretical analyses, and, consistent with scoping-review methodology, we did not critically appraise their quality; the synthesis therefore describes the range of descriptions in the literature and cannot weigh them by methodological quality. The sources also differ in their definitions of addiction, treatment and recovery, and most concern substance use disorders, with few on behavioral addictions; conclusions about behavioral addictions are therefore tentative. First-person descriptions were available only as quotations already selected by the authors of the primary studies, and two of them come from published autobiographical accounts rather than research participants (Table S5). Finally, the accounts of active addiction and of recovery come largely from different participants, so the comparison between stages is cross-sectional. The clinical vignette in Section 4.3 is a single anecdotal case from one author’s practice. It illustrates the framework but was not part of the evidence synthesized, and it cannot support claims about treatment effects.

The synthesis itself involved interpretation. One author carried out all coding without independent coding or inter-rater reliability assessment, and the selection and grouping of tags involved some subjectivity; the resulting categories should be treated as a guide to the discourse on temporality in addiction rather than a definitive classification. To integrate heterogeneous vocabularies, the synthesis focused on the past, present, and future and set aside finer phenomenological distinctions, such as those between layers of temporal experience or between temporal flow and structure. In addition, two authors have published work included in the review, which may have influenced the interpretation of their own sources.

Lastly, the review analyzes how temporal experience is described, not temporal experience itself. Changes in the language used in recovery may reflect genuine changes in experience, but also improved cognitive functioning during abstinence, adoption of therapeutic or recovery-community vocabulary, or social desirability (Section 3.4). The present data cannot distinguish between these explanations.

## 5. Conclusions

This scoping review suggests that first-person and professional accounts describe temporal experience in addiction in a largely consistent way: active addiction is characterized by a narrowed, repetitive present, an inaccessible or fragmented past and a foreshortened future, whereas recovery is associated with renewed access to the past, a more structured present and a wider, more concrete future. The two perspectives differ mainly in vocabulary, and first-person accounts reveal aspects of temporal experience, such as the unavailability rather than the negative valence of the past, and a compulsive rather than hedonistic present, which dispositional measures of time perspective do not capture.

Clinically, attention to how patients experience and describe time may help clinicians understand behaviors often judged in moral terms and structure treatment to re-establish continuity between the past, present, and future (Section 4.3; Table 3). These conclusions, however, are hypothesis-generating. The evidence is heterogeneous, was not appraised for quality, and is largely cross-sectional. The apparent convergence of patients’ and professionals’ vocabularies in recovery may reflect the adoption of therapeutic language rather than a change in experience. Longitudinal qualitative studies following the same individuals through treatment and recovery, comparisons across substances, behavioral addictions, age groups and cultures, and empirical tests of whether temporal descriptions track clinical change are needed before these findings can inform practice as markers of recovery.

## Figures and Tables

**Table 1:** Professional terms describing temporal experience in addiction and recovery, by temporal dimension. Numbers in brackets refer to the sources in which each term occurs.

Dimension	Addiction	Recovery
**Past**	lost time [27,28], fragmented [29], negative [30], inaccessible [31], loss of memory [27,28,32]	looking backward [25], new projection of self [10], re-evaluated [24], increased emphasis [24,31,33,34], increased positive attitude [11], rewritten [35]
**Present**	frozen [10,25,36], immediate [5,37,38,39,40], short temporal window [30,39,41,42,43,44], liquid [10,36,45], anthropological hyperpresentification [23,33], present continuous [43,45], atemporalization [46], eternal [46,47,48], oppressive dimension [42], more prevalent [49], no continuity [27,35,46], stuck [29,42], extended [50]	less orientation [51,52], shift in time focus [53], cut into more achievable pieces [53], less focus [23,53], reduced intensity [31,33]
**Future**	shifting from future to present [51,54], disappearance of long-term goals [30,55], closed [24,29,30], distant [29,38], less structured [11], lack of delayed gratification [2,56], low orientation [37,49,51,57], decreased sensitivity [5,57], dissociated [10]	moving forward [25], more orientation [12,38,49,51,52], augmented/extended [23,58], borrowed [43], increased emphasis [24,31,33,49]

**Table 2:** First-person descriptions of temporal experience in addiction and recovery, by temporal dimension (selection). Addiction type in parentheses: A, alcohol; C, cannabis; O, opioids/heroin; St, stimulants (crack cocaine); D, drugs (substance not specified or several substances); I, internet use. Supplementary Table S5 provides the complete set of quotations, with source, addiction type, and setting.

Dimension	Addiction	Recovery
**Past**	“I have a big hole in my memory, there are several months I cannot remember” (D) [59]; “There’s bits of it you can’t even remember (…) When you usin’ you’ve got no concept of time passin’” (O) [54]; “At that particular time I didn’t put it as making the inadequacy go away; all I knew is that it felt better” (A) [60]; “I liked football and wanted to be a personal trainer (…) but I changed my mind” (C) [61]; “Do the same thing; we never used to do anything else” (C) [62]; “I say that I’m gonna get blackout, but I really just mean I wanna go out and drink a lot and have fun, but I don’t necessarily wanna lose my memory” (A) [32]	“And I’m remembering things” (A) [63]; “I used to live like, I will go drinking next week, or think, I messed up my life so much in the past but (…) is just for today, and live in the moment” (A) [64]; “I’d had enough of using drugs (…) I’d had enough of not having enough money” (D) [65]; “I have the experience to tell what I should change in my life” (O) [66]; “My purpose was to find myself again, I want to feel all that good energy again” (D) [67]; “When I look back at the past, all the years of my life that I have wasted in my addiction…” (D) [68]
**Present**	“You don’t notice time at all” (D) [25]; “You recall that soft, pleasant feeling of relaxing and escaping, getting a little time-out” (A) [69]; “Sick every day” (A) [63]; “The solution was a temporary escape, and at times temporary was good enough” (D) [70]; “…that was basically what my day was all about, getting up and getting myself stoned so I could go to bed again” (C) [59]; “Because there’s no limit; there’s no mound; there’s no ending man. It’s the never-ending dream” (St) [48]; “It just depends on what I’m doing, if I’ve got a fair bit to do that can be a couple of hours” (I) [71]	“After treatment, I feel I have more time” (D) [68]; “I’m very happy with my life today and very happy with how things are going” (A) [60]; “It’s just for today” (D) [53]; “It is almost a euphoric experience to see that my daily life is ok without alcohol” (A) [69]; “I suppose it [practicing recovery] is just habitual as well now (…) it’s just part of my routine” (D) [72]; “You have to twist and turn your thinking (…) It takes time, and it’s a process” (D) [34]; “My ‘recovery’ has now become a phrase I am less inclined to use a great deal as it seems to indicate I am or was in some way ill” (A) [64]
**Future**	“It doesn’t matter about the next day” (O) [54]; “I will feel much more calm” (D) [73]; “Basically I took it [heroin] to stop me thinkin’ about the future” (O) [54]; “Actually, I have nothing to do, and so I smoke. I smoke in lack of better things to do” (C) [59]; “I do not see myself in a year’s time. I live from day to day (…) it’s impossible to say” (D) [43]	“I know that I have a future” (D) [74]; “Worried about the future (…) It’s very hard, it’s very energy consuming” (A) [64]; “It’s all openin’ up an’ the future’s gettin’ brighter an’ wider” (O) [54]; “And this gave me the strength to go on and that there is hope” (O) [66]; “Starting off with the AA (…) there’s this huge world for me” (D) [72]; “I want to become a biochemist” (D) [67]; “I don’t tend to worry about the future anymore” (D) [68]

**Table 3:** Suggestions for addressing temporal experience across phases of treatment, derived from the synthesis. The suggestions are hypothesis-generating and have not been tested as a protocol; numbers in brackets refer to the sources on which each suggestion draws.

Phase of Treatment	Temporal Pattern (Section 3)	Suggested Clinical Focus	Examples of Practices
**Assessment and engagement**	Past inaccessible or fragmented; present narrowed; future foreshortened	Explore how the patient experiences each temporal dimension; listen for temporal metaphors	Questions about the past, an ordinary day and expectations for the future; TATE [1] or ZTPI [6], repeated at discharge
**Early treatment and stabilization**	Present cyclic and organized around obtaining and using the substance	Replace the substance-centered routine with an external daily structure; divide the present into achievable units	Structured daily schedule [68]; short, achievable daily goals [53]; explaining the temporal rationale of structure to patients [49]
**Psychotherapy**	Past fragmented; discontinuous sense of self; future closed or distant	Reconnect past, present and future; widen and concretize the future	Developmental-history and narrative work [24,30]; re-evaluating past decisions [77]; episodic future thinking [39]; mindfulness-based interventions [78]
**Relapse prevention and aftercare**	Return of a present-centered pattern and loss of future plans during relapse	Recognize a renewed narrowing of the present as a warning sign; maintain concrete plans	Discussing early warning signs drawn from relapse accounts [69]; concrete educational or work plans [67]; monitoring future orientation [51,52]

## Data Availability

This study did not create or analyze new data. All material discussed is contained in the published articles listed in the reference list. The search strategy, the characteristics of the included sources, and the complete set of charted first-person descriptions are provided in Supplementary Tables S3–S5.

## Supplementary Materials

The following supporting information can be downloaded at https://www.mdpi.com/article/10.3390/jcm15218421/s1, Table S1: PRISMA-ScR checklist; Table S2: PRISMA 2020 checklist; Table S3: Search strategy: keyword combinations and number of records retrieved per source; Table S4: Characteristics of the included sources (*n* = 59); Table S5: Complete set of first-person descriptions of temporal experience, with source, type of addiction and setting. References [21,22] and the included sources listed in Table S4 are cited in the Supplementary Materials; all are included in the reference list of this article.

## Abbreviations

The following abbreviations are used in this manuscript:

ICD-11	International Classification of Diseases, 11th Revision
JBI	Joanna Briggs Institute
MeSH	Medical Subject Headings
PCC	Population–Concept–Context
PRISMA	Preferred Reporting Items for Systematic Reviews and Meta-Analyses
PRISMA-ScR	PRISMA extension for Scoping Reviews
TATE	Transdiagnostic Assessment of Temporal Experience
ZTPI	Zimbardo Time Perspective Inventory
