# Supplementary material for: Temporal Experience in Addiction and Recovery: A Scoping Review and Conceptual Synthesis of First-Person and Professional Accounts
Source: J Clin Med. 2026 Oct 29;15(21):8421. doi: 10.3390/jcm15218421 (PMC13764607; doi:10.3390/jcm15218421)
Supplement: Supplementary file 1 [file jcm-15-08421-s001.zip › Table_S1_PRISMA-ScR_checklist.pdf]

Supplementary Materials

Temporal Experience in Addiction and Recovery: A Scoping Review and Conceptual Synthesis of First-Person and Professional Accounts

Table S1. PRISMA-ScR checklist.

Section	Item	Checklist item	Reported in
TITLE	1	Title	Title (identified as a scoping review)
ABSTRACT	2	Structured summary	Abstract
INTRODUCTION	3	Rationale	Sections 1.1–1.3
INTRODUCTION	4	Objectives	Section 1.3 (Section 2.2 for population, concept and context)
METHODS	5	Protocol and registration	Section 2.1 (no review protocol was prepared or registered)
METHODS	6	Eligibility criteria	Section 2.2
METHODS	7	Information sources	Section 2.3; Table S3
METHODS	8	Search	Section 2.3; Table S3
METHODS	9	Selection of sources of evidence	Section 2.4
METHODS	10	Data charting process	Section 2.5
METHODS	11	Data items	Section 2.5; Table S4
METHODS	12	Critical appraisal of individual sources of evidence	Section 2.5 (not performed; rationale given)
METHODS	13	Synthesis of results	Section 2.6; Figure 2
RESULTS	14	Selection of sources of evidence	Section 2.4; Figure 1
RESULTS	15	Characteristics of sources of evidence	Section 3.1; Table S4
RESULTS	16	Critical appraisal within sources of evidence	Not applicable (no critical appraisal performed; Section 2.5)
RESULTS	17	Results of individual sources of evidence	Sections 3.2–3.3; Tables 1 and 2; Table S5
RESULTS	18	Synthesis of results	Sections 3.2–3.4; Tables 1 and 2
DISCUSSION	19	Summary of evidence	Section 4.1
DISCUSSION	20	Limitations	Section 4.4
DISCUSSION	21	Conclusions	Sections 4.2, 4.3 (Table 3) and 5
FUNDING	22	Funding	Funding statement: National Science Center, Poland (grant 2021/42/E/HS1/00106); the funder had no role in the review. Funding of included sources was not charted.

Items as listed in Tricco et al. (2018), PRISMA Extension for Scoping Reviews (PRISMA-ScR), Ann. Intern. Med. 169, 467–473. Locations refer to sections, tables and figures of the main manuscript.
