# Supplementary material for: Temporal Experience in Addiction and Recovery: A Scoping Review and Conceptual Synthesis of First-Person and Professional Accounts
Source: J Clin Med. 2026 Oct 29;15(21):8421. doi: 10.3390/jcm15218421 (PMC13764607; doi:10.3390/jcm15218421)
Supplement: Supplementary file 1 [file jcm-15-08421-s001.zip › Table_S2_PRISMA_2020_checklist.pdf]

Supplementary Materials

Temporal Experience in Addiction and Recovery: A Scoping Review and Conceptual Synthesis of First-Person and Professional Accounts

Table S2. PRISMA 2020 checklist.

Section and topic	Item	Reported in
Title	1	Title (identified as a scoping review)
Abstract	2	Abstract
Rationale	3	Sections 1.1–1.3
Objectives	4	Section 1.3
Eligibility criteria	5	Section 2.2
Information sources	6	Section 2.3
Search strategy	7	Section 2.3; Table S3
Selection process	8	Section 2.4
Data collection process	9	Section 2.5
Data items	10a	Sections 2.5–2.6
Data items	10b	Section 2.5; Table S4
Study risk of bias assessment	11	Not performed (scoping review); Section 2.5
Effect measures	12	Not applicable (no quantitative synthesis)
Synthesis methods	13a	Section 2.6
Synthesis methods	13b	Not applicable
Synthesis methods	13c	Section 2.6; Tables 1–2; Figure 2
Synthesis methods	13d	Section 2.6
Synthesis methods	13e	Not applicable
Synthesis methods	13f	Not applicable
Reporting bias assessment	14	Not applicable; see Section 4.4
Certainty assessment	15	Not applicable
Study selection	16a	Section 2.4; Figure 1
Study selection	16b	Section 2.4; Figure 1 (reasons only; counts not recorded)
Study characteristics	17	Section 3.1; Table S4
Risk of bias in studies	18	Not applicable (no risk-of-bias appraisal)
Results of individual studies	19	Tables 1–2; Table S5 (no effect estimates)
Results of syntheses	20a	Sections 3.1–3.4

Section and topic	Item	Reported in
Results of syntheses	20b	Not applicable
Results of syntheses	20c	Not applicable
Results of syntheses	20d	Not applicable
Reporting biases	21	Not applicable
Certainty of evidence	22	Not applicable
Discussion	23a	Section 4.1
Discussion	23b	Section 4.4
Discussion	23c	Section 4.4
Discussion	23d	Sections 4.2, 4.3 (Table 3) and 5
Registration and protocol	24a	Section 2.1 (not registered)
Registration and protocol	24b	Section 2.1 (no protocol prepared)
Registration and protocol	24c	Not applicable
Support	25	Funding statement
Competing interests	26	Conflicts of Interest statement
Availability of data, code and other materials	27	Data Availability Statement; Tables S3–S5

Items as listed in Page et al. (2021), The PRISMA 2020 statement, BMJ 372, n71. Items not applicable to a scoping review without quantitative synthesis are marked as such.
