# Supplementary material for: Temporal Experience in Addiction and Recovery: A Scoping Review and Conceptual Synthesis of First-Person and Professional Accounts
Source: J Clin Med. 2026 Oct 29;15(21):8421. doi: 10.3390/jcm15218421 (PMC13764607; doi:10.3390/jcm15218421)
Supplement: Supplementary file 1 [file jcm-15-08421-s001.zip › Table_S3_Search_strategy.pdf]

Supplementary Materials

Temporal Experience in Addiction and Recovery: A Scoping Review and Conceptual Synthesis of First-Person and Professional Accounts

Table S3. Search strategy: keyword combinations and number of records retrieved per source.

Searches were conducted between November 2023 and February 2024 without date restrictions or filters, using free-text terms; PubMed and Scopus were searched in titles, abstracts and keywords; Google Scholar with default settings. “Screened” indicates the 22 combinations whose records were screened (PubMed: 224 records; Scopus: 299 records; Google Scholar: first 100 records of the marked combination).

No.	Keyword combination	Source	Records retrieved	Screened
1	addiction	PubMed	13,403	No
2	addiction	Google Scholar	562,000	No
3	addiction	Scopus	151,287	No
4	addicted	PubMed	134,027	No
5	addicted	Google Scholar	162,000	No
6	addicted	Scopus	9,550	No
7	"substance abuse"	PubMed	26,054	No
8	"substance abuse"	Google Scholar	691,000	No
9	"substance abuse"	Scopus	119,326	No
10	addiction AND time	PubMed	22,737	No
11	addiction AND time	Google Scholar	17,800	No
12	addiction AND time	Scopus	25,885	No
13	addicted AND time	PubMed	22,737	No
14	addicted AND time	Google Scholar	17,800	No
15	addicted AND time	Scopus	1,854	No
16	addiction AND "time perception"	PubMed	615	No
17	addiction AND "time perception"	Google Scholar	17,900	No
18	addiction AND "time perception"	Scopus	108	No
19	addicted AND "time perception"	PubMed	615	No
20	addicted AND "time perception"	Google Scholar	17,900	No
21	addicted AND "time perception"	Scopus	7	No
22	addiction AND "time flow"	PubMed	161	No
23	addiction AND "time flow"	Google Scholar	26,500	No
24	addiction AND "time flow"	Scopus	1	No
25	addicted AND "time flow"	PubMed	161	No

No.	Keyword combination	Source	Records retrieved	Screened
26	addicted AND "time flow"	Google Scholar	18,200	No
27	addicted AND "time flow"	Scopus	0	No
28	addiction AND "lived time"	PubMed	1,106	No
29	addiction AND "lived time"	Google Scholar	17,900	No
30	addiction AND "lived time"	Scopus	5	No
31	addicted AND "lived time"	PubMed	1,106	No
32	addicted AND "lived time"	Google Scholar	17,900	No
33	addicted AND "lived time"	Scopus	1	No
34	addiction AND synchrony	PubMed	58	No
35	addiction AND synchrony	Google Scholar	16,100	No
36	addiction AND synchrony	Scopus	43	No
37	addicted AND synchrony	PubMed	58	No
38	addicted AND synchrony	Google Scholar	16,100	No
39	addicted AND synchrony	Scopus	2	No
40	addiction AND temporal*	PubMed	2,197	No
41	addiction AND temporal*	Google Scholar	36,300	No
42	addiction AND temporal*	Scopus	2,060	No
43	addicted AND temporal*	PubMed	2,197	No
44	addicted AND temporal*	Google Scholar	36,300	No
45	addicted AND temporal*	Scopus	120	No
46	addiction AND "temporal experience"	PubMed	142	No
47	addiction AND "temporal experience"	Google Scholar	24,000	No
48	addiction AND "temporal experience"	Scopus	16	Yes
49	addiction AND time AND phenomenology	PubMed	37	Yes
50	addiction AND time AND phenomenology	Google Scholar	20,700	No
51	addiction AND time AND phenomenology	Scopus	78	Yes
52	addicted AND time AND phenomenology	PubMed	37	Yes
53	addicted AND time AND phenomenology	Google Scholar	20,700	No
54	addicted AND time AND phenomenology	Scopus	5	Yes
55	addiction AND "temporal experience" AND phenomenology	PubMed	2	No
56	addiction AND "temporal experience" AND phenomenology	Google Scholar	17,300	No

No.	Keyword combination	Source	Records retrieved	Screened
57	addiction AND "temporal experience" AND phenomenology	Scopus	3	Yes
58	addicted AND "temporal experience" AND phenomenology	PubMed	2	Yes
59	addicted AND "temporal experience" AND phenomenology	Google Scholar	17,300	No
60	addicted AND "temporal experience" AND phenomenology	Scopus	0	No
61	addiction AND temporal* AND phenomenology	PubMed	11	Yes
62	addiction AND temporal* AND phenomenology	Google Scholar	17,200	No
63	addiction AND temporal* AND phenomenology	Scopus	20	Yes
64	addicted AND temporal* AND phenomenology	PubMed	11	Yes
65	addicted AND temporal* AND phenomenology	Google Scholar	17,200	No
66	addicted AND temporal* AND phenomenology	Scopus	2	Yes
67	addiction AND time AND first-person	PubMed	12	Yes
68	addiction AND time AND first-person	Google Scholar	18,400	No
69	addiction AND time AND first-person	Scopus	18	Yes
70	addicted AND time AND first-person	PubMed	12	Yes
71	addicted AND time AND first-person	Google Scholar	18,400	No
72	addicted AND time AND first-person	Scopus	18	Yes
73	addiction AND temporal* AND first-person	PubMed	3	Yes
74	addiction AND temporal* AND first-person	Google Scholar	17,100	No
75	addiction AND temporal* AND first-person	Scopus	2	Yes
76	addicted AND temporal* AND first-person	PubMed	3	Yes
77	addicted AND temporal* AND first-person	Google Scholar	17,100	No
78	addicted AND temporal* AND first-person	Scopus	0	No
79	addiction AND time* AND phenomenolog*	PubMed	96	Yes
80	addiction AND time* AND phenomenolog*	Google Scholar	22,200	Yes
81	addiction AND time* AND phenomenolog*	Scopus	125	Yes
82	addicted AND time* AND phenomenolog*	PubMed	96	No
83	addicted AND time* AND phenomenolog*	Google Scholar	22,200	No
84	addicted AND time* AND phenomenolog*	Scopus	12	Yes
