# Supplementary material for: Temporal Experience in Addiction and Recovery: A Scoping Review and Conceptual Synthesis of First-Person and Professional Accounts
Source: J Clin Med. 2026 Oct 29;15(21):8421. doi: 10.3390/jcm15218421 (PMC13764607; doi:10.3390/jcm15218421)
Supplement: Supplementary file 1 [file jcm-15-08421-s001.zip › Table_S4_Characteristics_of_sources.pdf]

Supplementary Materials

Temporal Experience in Addiction and Recovery: A Scoping Review and Conceptual Synthesis of First-Person and Professional Accounts

Table S4. Characteristics of the included sources (n = 59).

Ref.: reference number in the main manuscript. Type of source: classification by main approach (Section 2.5). Perspective: type of description contributed to the synthesis. † includes first-person material from published autobiographical accounts.

Ref.	Source	Type of source	Design	Type of addiction	Population/setting	Perspective	Stage
2	Wittmann et al., 2007	Psychological	Experimental (time perception and motor timing tasks)	Stimulants	Stimulant-dependent adults and controls	Professional	Addiction
5	Petry et al., 1998	Psychological	Quantitative (time horizon, delay discounting)	Opioids (heroin)	Heroin-dependent adults and controls	Professional	Addiction
10	Moskalewicz, 2016	Phenomenological	Phenomenological analysis	Drugs (several)	People with addiction in treatment	Professional	Addiction, recovery
11	Hulbert & Lens, 1988	Psychological	Review	Alcohol	–	Professional	Addiction, recovery
12	Wills et al., 2001	Psychological	Quantitative (survey)	Substances (tobacco, alcohol, cannabis)	Adolescents	Professional	Addiction
23	Messas & Tamelini, 2021	Phenomenological	Theoretical (book chapter)	Substance misuse	–	Professional	Addiction, recovery
24	Kemp, 2019	Interview	Qualitative (phenomenological)	Drugs and alcohol	People in recovery	First-person, professional	Addiction, recovery
25	Roe et al., 2023	Interview	Qualitative (ethnography)	Heroin and polysubstance use	People who use drugs	First-person, professional	Addiction
27	Zakrzewski & Hector, 2004	Interview	Qualitative (phenomenological)	Alcohol	Men in Alcoholics Anonymous	First-person	Addiction, recovery
28	Kvamme et al., 2015	Interview	Qualitative (phenomenological-hermeneutic)	Alcohol	Adults who relapsed after rehabilitation	First-person	Addiction, recovery
29	Rodriguez-Morales, 2019	Interview	Qualitative	Alcohol and drugs	People in twelve-step recovery	First-person	Addiction, recovery
30	Dörr et al., 2014	Interview	Qualitative	Cannabis	Adolescent habitual users	First-person	Addiction
31	Salmon & Wohl, 2020	Psychological	Mixed methods	Addiction (various)	People living with addiction	First-person, professional	Addiction
32	Goodwin et al., 1969	Psychological	Clinical interview study	Alcohol	Hospitalized patients with alcoholism	Professional	Addiction
33	Miller et al., 2018	Interview	Mixed methods	Alcohol	Young adults reporting blackouts	First-person, professional	Addiction
34	Erdős et al., 2009	Intervention	Qualitative (group therapy)	Drugs (incl. opioids)	Drug-dependent persons in recovery	First-person, professional	Addiction, recovery
35	Rose & Grant, 2010	Psychological	Review	Alcohol	–	Professional	Addiction
36	Nigro et al., 2019	Psychological	Quantitative	Gambling	Adults with gambling disorder	Professional	Addiction
37	Rochat et al., 2019	Psychological	Theoretical review	Addiction (general)	–	Professional	Addiction

Ref.	Source	Type of source	Design	Type of addiction	Population/setting	Perspective	Stage
38	Noël et al., 2017	Psychological	Review	Addictive behaviors (general)	–	Professional	Addiction
39	Smith, 1998	Interview	Qualitative (hermeneutic phenomenology)	Alcohol	Problem drinkers	First-person	Addiction, recovery
40	Kemp, 2009	Phenomenological	Theoretical	Drugs (general)	–	Professional	Addiction, recovery
41	Järvinen & Ravn, 2017	Interview	Qualitative	Drugs (mainly cannabis)	Young people in drug treatment	First-person, professional	Addiction
42	Mørck et al., 2020	Interview	Qualitative (reflective lifeworld research)	Alcohol	Outpatients in alcohol treatment	First-person	Addiction
43	Hilte, 2019	Phenomenological	Qualitative (biographical/narrative material)	Heroin, cannabis	Drug users; published autobiographical accounts	First-person (†), professional	Addiction
44	Trujillo, 2004	Interview	Qualitative (existential-phenomenological)	Crack cocaine	People using crack cocaine	First-person, professional	Addiction
45	Copoeru, 2018	Phenomenological	Theoretical	Addiction (general)	–	Professional	Addiction
46	Moskalewicz & Schwartz, 2020	Phenomenological	Theoretical	Mental disorders including addiction	–	Professional	Addiction, recovery
47	Copoeru, 2014	Phenomenological	Theoretical	Addiction (general)	–	Professional	Addiction
48	Di Petta, 2014a	Phenomenological	Theoretical/clinical	Drugs (incl. heroin)	–	Professional	Addiction
49	Messas, 2014	Phenomenological	Theoretical	Alcohol	–	Professional	Addiction
50	Warren, 2018	Interview	Qualitative (narrative analysis)	Substance misuse	People in recovery	First-person	Addiction, recovery
51	Moskalewicz & Messas, 2022	Phenomenological	Theoretical/clinical	Substance misuse	–	First-person, professional	Addiction, recovery
52	Rafei et al., 2021	Intervention	Theoretical (intervention proposal)	Substance use disorders	–	Professional	Addiction, recovery
53	Klingemann, 2001	Interview	Qualitative	Alcohol and drugs	Patients and staff of treatment clinics	Professional	Addiction, recovery
54	Kemp, 2020	Phenomenological	Theoretical (with case material)	Drugs (general)	–	First-person, professional	Addiction, recovery
55	Sweet, 2012	Phenomenological	Theoretical (psychoanalytic)	Addiction (general)	–	Professional	Addiction
56	Besana, 2026	Phenomenological	Book review	Substance misuse	–	Professional	Addiction, recovery
57	Hanemaayer, 2010	Phenomenological	Theoretical	Addiction (general)	–	Professional	Recovery
58	Kin, 2021	Interview	Qualitative (IPA; doctoral dissertation)	Drugs	Ex-addicts and ex-offenders	First-person	Recovery
59	Lennings, 1996	Psychological	Quantitative (treatment outcome)	Alcohol	Severely dependent patients in treatment	Professional	Addiction, recovery
60	Chodkiewicz, 2011	Psychological	Quantitative (ZTPI)	Alcohol	Patients in alcohol treatment	Professional	Addiction, recovery
61	Rodriguez & Smith, 2014	Interview	Qualitative (IPA)	Alcohol and drugs	Young men in early recovery	First-person, professional	Recovery
62	Messas et al., 2019	Intervention	Theoretical (treatment principles)	Substance misuse	–	Professional	Addiction, recovery

Ref.	Source	Type of source	Design	Type of addiction	Population/setting	Perspective	Stage
63	Reith, 1999	Interview	Qualitative	Heroin	People using heroin	First-person, professional	Addiction, recovery
64	Pérez Vázquez et al., 2018	Interview	Qualitative (phenomenological)	Drugs	Adolescents	First-person	Addiction, recovery
65	Alvos et al., 1993	Psychological	Quantitative	Injecting drugs	Current and former injecting drug users	Professional	Addiction, recovery
66	Davies & Filippopoulos, 2015	Intervention	Mixed methods	Substances (several)	Clients in residential treatment	First-person, professional	Addiction, recovery
67	Hilte, 2022	Interview	Qualitative (narrative)	Drugs	People in recovery	First-person, professional	Recovery
68	Di Petta & Tittarelli, 2019	Intervention	Clinical/theoretical (group therapy)	Drugs	–	Professional	Recovery
69	Kemp, 2022	Phenomenological	Theoretical	Addiction (general)	–	Professional	Addiction
70	Paasche et al., 2019	Psychological	Review	Alcohol, tobacco, cannabis, cocaine, gambling	–	Professional	Addiction
71	Di Petta, 2014b	Phenomenological	Theoretical	Drugs	–	Professional	Addiction
72	Smart, 1968	Psychological	Quantitative	Alcohol	Patients with alcoholism and social drinkers	Professional	Addiction
73	Schlimme, 2010	Phenomenological	Theoretical (with autobiographical account)	Opioids	–	First-person (†), professional	Addiction
74	Cope, 2003	Interview	Qualitative	Cannabis, heroin	Young male prisoners	First-person	Addiction
75	Banonis, 1989	Interview	Qualitative (phenomenological)	Drugs	People recovering from addiction	First-person	Addiction, recovery
76	Johnson & Keane, 2017	Interview	Qualitative	Internet use	Internet users	First-person, professional	Addiction
77	Shinebourne & Smith, 2011	Interview	Qualitative (IPA)	Alcohol and drugs	People in long-term recovery	First-person	Recovery
