# Supplementary material for: Temporal Experience in Addiction and Recovery: A Scoping Review and Conceptual Synthesis of First-Person and Professional Accounts
Source: J Clin Med. 2026 Oct 29;15(21):8421. doi: 10.3390/jcm15218421 (PMC13764607; doi:10.3390/jcm15218421)
Supplement: Supplementary file 1 [file jcm-15-08421-s001.zip › Table_S5_First-person_descriptions.pdf]

Supplementary Materials

Temporal Experience in Addiction and Recovery: A Scoping Review and Conceptual Synthesis of First-Person and Professional Accounts

Table S5. Complete set of first-person descriptions of temporal experience.

Type of addiction: A, alcohol; C, cannabis; O, opioids/heroin; St, stimulants (crack cocaine); D, drugs (substance not specified or several substances); I, internet use. † Published autobiographical account quoted in the source, not a research participant. Quotations are reproduced verbatim from the sources; (…) marks omitted text. Numbers in brackets refer to the reference list of the main manuscript.

No.	Dimension	Stage	Quotation	Source [ref.]	Type
1	Past	Addiction	"you can't remember"	Roe et al., 2023 [25]	D
2	Past	Addiction	"I have no recollection of that period of time"	Kemp, 2020 [54]	D
3	Past	Addiction	"At that particular time I didn't put it as making the inadequacy go away; all I knew is that it felt better."	Zakrzewski & Hector, 2004 [27]	A
4	Past	Addiction	"I was a person full of life"	Salmon & Wohl, 2020 [31]	D
5	Past	Addiction	"There's bits of it you can't even remember (…) When you usin you've got no concept of time passin'"	Reith, 1999 [63]	O
6	Past	Addiction	"I liked football and wanted to be a personal trainer physical education teacher or any of those things, but I changed my mind"	Dörr et al., 2014 [30]	C
7	Past	Addiction	"I have a big hole in my memory, there are several months I cannot remember"	Järvinen & Ravn, 2017 [41]	D
8	Past	Addiction	"Do the same thing; we never used to do anything else"	Cope, 2003 [74]	C
9	Past	Addiction	"I am drunk—I have been drinking, and I can't help it. I've lost it completely"	Mørck et al., 2020 [42]	A
10	Past	Addiction	"I've definitely had that experience, where it's like—I say that I'm gonna get blackout, but I really just mean I wanna go out and drink a lot and have fun, but I don't necessarily wanna lose my memory"	Miller et al., 2018 [33]	A
11	Past	Recovery	"it's so much better than the past"	Kin, 2021 [58]	D
12	Past	Recovery	"And I'm remembering things"	Smith, 1998 [39]	A
13	Past	Recovery	"I used to live like, I will go drinking next week, or think, I messed up my life so much in the past but, you know, is just for today, and live in the moment."	Rodriguez-Morales, 2019 [29]	A
14	Past	Recovery	"I'd had enough of using drugs, I'd had enough of waking up stinking of piss. I'd had enough of not having enough money"	Warren, 2018 [50]	D
15	Past	Recovery	"I have the experience to tell what I should change in my life. I first used poppy tea when I was 15, and I think have become addicted since 12"	Erdős et al., 2009 [34]	O
16	Past	Recovery	"What I think about are situations that maybe occurred just on a not a flash or daily basis"	Banonis, 1989 [75]	D
17	Past	Recovery	"It would've been darker colours and feelings, sort of fear or regret or sort of hope and anticipation"	Shinebourne & Smith, 2011 [77]	D
18	Past	Recovery	"My purpose was to find myself again, I want to feel all that good energy again"	Pérez Vázquez et al., 2018 [64]	D
19	Past	Recovery	"To appreciate the little things, I couldn't do that before. I couldn't appreciate the little things."	Hilte, 2022 [67]	D
20	Past	Recovery	"When I look back at the past, all the years of my life that I have wasted in my addiction…"	Davies & Filippopoulos, 2015 [66]	D
21	Present	Addiction	"You don't notice time at all"	Roe et al., 2023 [25]	D
22	Present	Addiction	"You recall that soft, pleasant feeling of relaxing and escaping, getting a little time-out"	Kvamme et al., 2015 [28]	A
23	Present	Addiction	"Sick every day"	Smith, 1998 [39]	A
24	Present	Addiction	"Everything has become harder for me"	Salmon & Wohl, 2020 [31]	D

No.	Dimension	Stage	Quotation	Source [ref.]	Type
25	Present	Addiction	"The solution was a temporary escape, and at times temporary was good enough. I did not know how to deal with the emotional"	Banonis, 1989 [75]	D
26	Present	Addiction	"I am more focused on smoking a joint than on earning money […] I told them [the treatment centre] that I smoked 15–20 joints a day, and that was basically what my day was all about, getting up and getting myself stoned so I could go to bed again"	Järvinen & Ravn, 2017 [41]	C
27	Present	Addiction	"The way I look at it is it makes time go faster, cos there are times when you can't sleep and that I ain't got a job or nothing, I'm just in my cell all day so it's like I sleep all day"	Cope, 2003 [74]	C
28	Present	Addiction	"I also know that I will always bring myself to situations where I need to disconnect"	Mørck et al., 2020 [42]	A
29	Present	Addiction	"it just depends on what I'm doing, if I've got a fair bit to do that can be a couple of hours, if there's not quite so much to do and there's a bit of chatting going on."	Johnson & Keane, 2017 [76]	I
30	Present	Addiction	"I did absolutely nothing. I could look at the end of my shoes for eight hours. I was only aroused to action when the hourglass of junk ran out" †	Schlimme, 2010 [73]	O
31	Present	Addiction	"When I turned to heroin, I wanted to halt the flow of time, not so much out of a desire to remain young, but out of a fear of the injuries time might bring" †	Hilte, 2019 [43]	O
32	Present	Addiction	"Because there's no limit; there's no mound; there's no ending man. It's the never-ending dream. There is none"	Trujillo, 2004 [44]	St
33	Present	Addiction	"an hour's kind of a weird amount of time. Like you said, it's never just an hour. It's usually like, I can't really—can't put a number on it."	Miller et al., 2018 [33]	A
34	Present	Recovery	"After treatment, I feel I have more time"	Davies & Filippopoulos, 2015 [66]	D
35	Present	Recovery	"I'm very happy with my life today and very happy with how things are going"	Zakrzewski & Hector, 2004 [27]	A
36	Present	Recovery	"It's just for today"	Rodriguez & Smith, 2014 [61]	D
37	Present	Recovery	"My 'recovery' has now become a phrase I am less inclined to use a great deal as it seems to indicate I am or was in some way ill. I feel like a pretty balanced human being these days"	Rodriguez-Morales, 2019 [29]	A
38	Present	Recovery	"I think I'm now the person that I was always supposed to be"	Warren, 2018 [50]	D
39	Present	Recovery	"I'm in a different place now altogether. It is almost a euphoric experience to see that my daily life is ok without alcohol"	Kvamme et al., 2015 [28]	A
40	Present	Recovery	"I suppose it [practicing recovery] is just habitual as well now, it's kind of it's just part of my routine really ahm and it's the enabling part of my routine"	Shinebourne & Smith, 2011 [77]	D
41	Present	Recovery	"But you have to think a little bit further than I used to do. I didn't think that way, but I have changed the way I think. You have to twist and turn your thinking, which has been quite hard. It takes time, and it's a process."	Hilte, 2022 [67]	D
42	Present	Recovery	"Being in treatment has changed my understanding and experience of time, especially in mindfulness and the power of now."	Davies & Filippopoulos, 2015 [66]	D
43	Future	Addiction	"It doesn't matter about the next day"	Reith, 1999 [63]	O
44	Future	Addiction	"I will feel much more calm"	Salmon & Wohl, 2020 [31]	D
45	Future	Addiction	"Basically I took it [heroin] to stop me thinkin' about the future"	Reith, 1999 [63]	O
46	Future	Addiction	"Actually, I have nothing to do, and so I smoke. I smoke in lack of better things to do"	Järvinen & Ravn, 2017 [41]	C
47	Future	Addiction	"I do not see myself in a year's time. I live from day to day… I do not know, it's impossible to say."	Moskalewicz & Messas, 2022 [51]	D
48	Future	Recovery	"I know that I have a future"	Kin, 2021 [58]	D

No.	Dimension	Stage	Quotation	Source [ref.]	Type
49	Future	Recovery	"Worried about the future, what you may be thinking of me, you know how the world is. It's very hard, it's very energy consuming"	Rodriguez-Morales, 2019 [29]	A
50	Future	Recovery	"it's all openin' up an' the future's gettin' brighter an' wider"	Reith, 1999 [63]	O
51	Future	Recovery	"And this gave me the strength to go on and that there is hope"	Erdős et al., 2009 [34]	O
52	Future	Recovery	"Starting off with the AA, I think what I'm just trying to show is that there's this huge world for me"	Shinebourne & Smith, 2011 [77]	D
53	Future	Recovery	"I want to become a biochemist. I like chemistry a lot, I am more or less good at that"	Pérez Vázquez et al., 2018 [64]	D
54	Future	Recovery	"I don't tend to worry about the future anymore"	Davies & Filippopoulos, 2015 [66]	D
